# Teamwork Assessment Tools in Modern Surgical Practice: A Systematic Review

**DOI:** 10.1155/2015/494827

**Published:** 2015-09-03

**Authors:** George Whittaker, Hamid Abboudi, Muhammed Shamim Khan, Prokar Dasgupta, Kamran Ahmed

**Affiliations:** ^1^School of Medical Education, King's College London, London SE1 1UL, UK; ^2^Department of Urology, Guy's and St. Thomas' NHS Foundation Trust, London SE1 9RT, UK; ^3^MRC Centre for Transplantation, King's College London, London SE1 9RT, UK

## Abstract

*Introduction*. Deficiencies in teamwork skills have been shown to contribute to the occurrence of adverse events during surgery. Consequently, several teamwork assessment tools have been developed to evaluate trainee nontechnical performance. This paper aims to provide an overview of these instruments and review the validity of each tool. Furthermore, the present paper aims to review the deficiencies surrounding training and propose several recommendations to address these issues. *Methods*. A systematic literature search was conducted to identify teamwork assessment tools using MEDLINE (1946 to August 2015), EMBASE (1974 to August 2015), and PsycINFO (1806 to August 2015) databases. *Results*. Eight assessment tools which encompass aspects of teamwork were identified. The Nontechnical Skills for Surgeons (NOTSS) assessment was found to possess the highest level of validity from a variety of sources; reliability and acceptability have also been established for this tool. *Conclusions*. Deficits in current surgical training pathways have prompted several recommendations to meet the evolving requirements of surgeons. Recommendations from the current paper include integration of teamwork training and assessment into medical school curricula, standardised formal training of assessors to ensure accurate evaluation of nontechnical skill acquisition, and integration of concurrent technical and nontechnical skills training throughout training.

## 1. Introduction

Due to the sporadic and potentially catastrophic consequences of errors in surgery, the operating theatre environment has been described as a high-reliability organisation (HRO) [1]. Effective teamwork is a vital component of minimising human error and maintaining high reliability, hence the importance of enhancing nontechnical performance of surgical teams. Analysis of adverse events in surgery reveals deficiencies in nontechnical skills (NTS), the cognitive and interpersonal skills required for effective cooperation, as a major contributing factor to surgical errors [2]. These skills also have a direct impact on the technical performance of surgeons [3]. Furthermore, inadequate teamwork has been linked to a higher incidence of adverse events [4], whilst improvement of team-working ability with training correlates with reduced technical errors [5] and perioperative mortality [6]. This body of evidence highlights the importance of effective teamwork in surgery and, due to the inability of surgeons to accurately self-assess their level of this essential skill [7], the need for NTS assessment.

Surgical trainee performance is continually evaluated with a variety of validated tools. Technical skills assessments such as Procedure-Based Assessment (PBA) and Objective Structured Assessment of Technical Skills (OSATS) are used to measure procedural competence. NTS assessment tools are also used to scrutinise the generic interpersonal (teamwork, leadership, and communication) and cognitive (decision-making, situation awareness, and task management) qualities which complement technical skills [8]. Specific teamwork assessment tools aim to evaluate team-working ability by assessing observable behaviours using a skills taxonomy and behavioural marker system within several areas identified for successful teamwork such as communication and cooperation.

Developing tools for evaluating teamwork can be problematic due to the difficulty in quantifying inherently complex behaviours [9]. Like many other constructs, nontechnical surgical skills have no unique or specific criterion to predict outcomes, and the domain of content to sample is very broad and inclusive (e.g., respect, integrity, accountability, and communications). Before introduction, it must therefore be established as psychometrically robust with numerous sources of evidence confirming validity and reliability [10].

Validity represents the extent to which a test or assessment tool accurately measures the domains it has been designed to measure, whereas reliability refers to the ability of a test to produce consistent results. There are three main types of internal test validity (construct, content, and criterion), each of which can be subdivided. Legitimate proof for each of these attributes is required for a tool to be fully validated.

Construct validity is the extent to which a test measures the intended construct, demonstrated by linkages between expected and acquired measurements (e.g., participants with more experience achieving higher test scores). The linkages may be correlation-based (Pearson's *r*, regression, factor analysis, etc.) or experimentally based hypothesis testing specifying between group differences (analyses of variance, etc.). Convergent validity, the degree of relation between two similar constructs, and discriminant (divergent) validity, which concerns the degree of dissimilarity between two unrelated concepts, are the two subtypes construct validity.

Content validity concerns the extent to which a test represents all possible items in the domain being assessed and is subjectively determined by a group of experts with the appropriate background. Face validity is the participant's subjective estimate of whether the test appears to be effective at what it purports to be measuring.

Criterion validity relates to the accuracy of a test at predicting outcomes from other variables and is demonstrated by correlation of test scores with a criterion measure. There are two subtypes of criterion validity: concurrent and predictive. Concurrent validity is illustrated by correlation of test scores with simultaneous results from a previously validated tool that measures the same construct. Predictive validity is demonstrated when future test scores are accurately predicted following an initial assessment with a time period between the tests, although accurate prognostication of behaviours also constitutes evidence for predictive validity.

This paper aims to provide an overview of the teamwork assessment tools available for surgery and review the internal validation evidence supporting each instrument. Deficiencies of the current surgical training pathway will also be highlighted with subsequent formation of recommendations for training.

## 2. Methods

The Preferred Reporting Items for Systematic Reviews and Meta-Analyses (PRISMA) method was used as a guideline for the systematic review. A comprehensive literature search was conducted to identify teamwork assessment tools and supportive literature using MEDLINE (1946 to August 2015), EMBASE (1974 to August 2015), and PsycINFO (1806 to August 2015) databases. The following keywords were used in combination using the Boolean operators “OR” and “AND”: “teamwork,” “team work,” “team-working,” “non-technical skills,” “nontechnical skills,” “assessment,” “assessing,” “surgery,” “surgical,” and “surgeons.” The title of each study was screened for relevance, with retrieval of the abstract and full article if any doubt was present, followed by a selective process in which articles that failed to meet the criteria were excluded.

A wide variety of studies concerning teamwork assessment instruments were included. Original articles regarding the development process of specific instruments were used to identify the assessment tools. Validation studies were also included in addition to those which evaluated reliability and feasibility. Other articles which offered valuable information on teamwork assessment tools were included, encompassing studies which utilised the instruments for other development processes. Conference abstracts were also included.

Teamwork assessment tools developed for a specific surgical subspecialty were excluded because the authors' aim was to provide a broad overview of the general instruments available. Studies regarding assessment tools for technical skills were also excluded. Reviews, case reports, letters, and editorials were excluded in addition to articles in other languages. Duplicates were also removed.

## 3. Results

**Figure 1:**
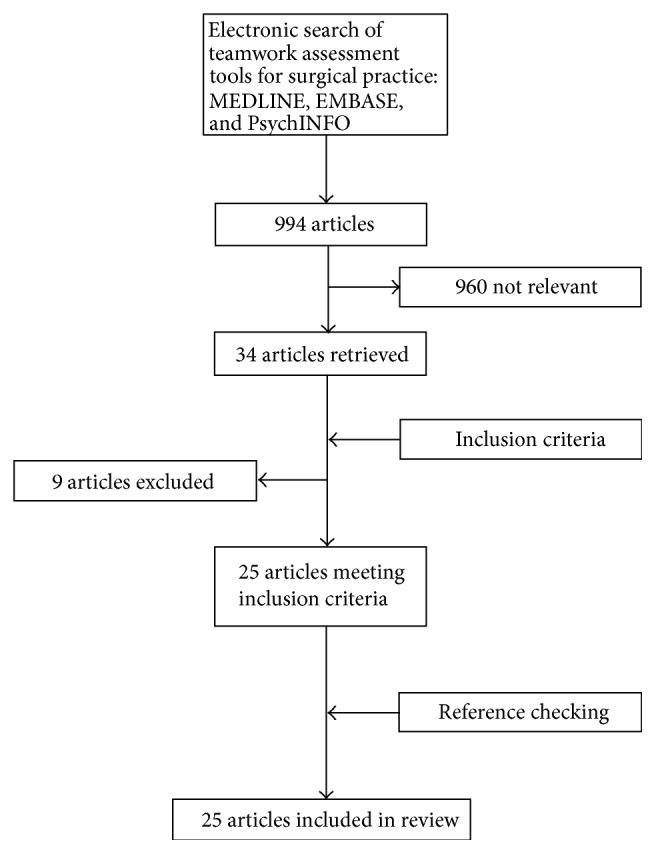
Flowchart depicting literature search strategy and results.

The literature search retrieved 994 publications and conference abstracts of potential relevance. Of these, 960 were determined as irrelevant and were subsequently excluded. Of the 34 articles remaining, further nine were excluded as they either failed to meet the inclusion criteria or fell under the exclusion criteria. After reference checking a final total of 25 articles were included in this review (Figure 1).

Eight teamwork assessment tools were identified from the search results generated (Table 1). These tools were developed for a range of healthcare professionals including surgeons, anaesthetists, and operating department practitioners. A total of 13 validation studies were included, some examples of which are presented in (Table 2). The amount and variety of validation evidence differed greatly between each tool.

## 4. Discussion

### 4.1. Observational Teamwork Assessment for Surgery (OTAS)

The OTAS tool, created from a generalised model of teamwork [24], assesses the NTS of the entire surgical team. Procedures are divided into three phases (preoperative, intraoperative, and postoperative), each of which incorporates three stages. In each stage, a psychologist rates behaviours observed in each theatre subteam (surgeons, anaesthetists, and nurses) against a list of exemplary conducts using a 7-point scale ranging from 0 (severely hindering team function) to 6 (greatly enhancing team function). Analysis of behaviours is implemented in five domains: communication, cooperation, coordination, shared leadership, and team monitoring and situation awareness. A generic checklist is employed simultaneously, with individual marks being awarded for each task completed in three categories (patient-related, equipment-related, and communication-related).

Construct validity was illustrated in an article which compared behavioural ratings given by an expert/expert pair and expert/novice pair of assessors for 12 surgical procedures [11]. Significant consistency was observed in the expert/expert pair for 12 of the 15 behaviours (*r*_*s*_ = 0.51–0.94, *p* < 0.05), whilst significant consistency was observed in the expert/novice pair for only 3 of the 15 behaviours (*r*_*s*_ = 0.52–0.60, *p* < 0.05). Exemplary behavioural markers were analysed in another article, wherein a panel of 15 experienced theatre practitioners (5 surgeons, 5 anaesthetists, and 5 scrub nurses) rated a substantial amount of markers as key factors of teamwork, indicating a high level of content validity [12]. Furthermore, two blinded assessors observed 30 operations to evaluate the observability of the exemplary behaviours provided, with high agreement (Cohen's *κ* ≥ 0.41) found for 84% of markers. In the second phase of this study, a group of three experts in nontechnical skills and patient safety reviewed each individual exemplar, resulting in the removal of 21 exemplars and the modification of further 23 markers.

OTAS is a robust tool for precisely assessing NTS due to inclusion of exemplar behaviours for reference, variety of nontechnical domains, and subdivision of procedure and staff. There is good evidence of construct and content validity. The instrument has also demonstrated high reliability [25] and feasibility [26] in other studies. However, intensive training of assessors is required [11] and determination of criterion validity (concurrent or predictive) is necessary.

### 4.2. Nontechnical Skills for Surgeons (NOTSS)

NOTSS is a behavioural rating system which aims to evaluate the intraoperative NTS of individual trainees. The tool was created by devising an appropriate skills taxonomy using a variation of the Delphi method in which consultant surgeons were interviewed about challenging emergency procedures they had performed, ensuring face validity [13]. An accompanying behavioural marker system was then developed to rate the identified skills within four constructs: situation awareness, decision-making, communication and teamwork, and leadership. Each domain is comprised of three elements which are numerically scored from 1 (poor) to 4 (good) (Table 3). A successive feedback session allows the trainees to reflect and develop their NTS for future procedures.

Multiple validities have been established for NOTSS. Concurrent validity was illustrated in a prospective observational study involving assessment of 85 surgical trainees throughout 404 procedures using NOTSS, PBA, and OSATS tools [15]. A total of 715 assessments were made by 100 staff members including anaesthetists, scrub nurses, surgical care practitioners, and independent assessors. Results showed significant positive correlations between NOTSS, PBA (*r* = 0.43–0.55, *p* < 0.001), and OSATS (*r* = 0.40–0.58, *p* < 0.001) in all four domains. In a similar experiment, 85 surgical trainees were rated by 148 assessors (including consultants, anaesthetists, nurses, surgical care practitioners, and independent assessors) across 437 cases [14]. NOTSS, PBA, and OSATS tools were employed simultaneously to produce 1635 completed assessments. Associations were observed between training grade and NOTSS scores (*r* = 0.40–0.57, *p* < 0.001), and within the four behavioural categories (*r* = 0.74–0.76, *p* < 0.001), demonstrating construct and content validities, respectively.

NOTSS has shown high levels of validity and acceptability in practice and has the potential to become a fundamental tool in surgical curricula due to its comprehensive coverage of NTS. Furthermore, interobserver reliability has been established [27], though assessors need a high level of training to obtain accurate results [28]. NOTSS therefore appears to be a valuable tool for the assessment of teamwork in surgery.

### 4.3. Oxford Nontechnical Skills System (NOTECHS)

NOTECHS is an evaluation tool which has been translated from the aviation industry to surgical practice via expert consultation and task analysis [16]. It is used to assess the nontechnical performance of surgical teams in four areas: leadership and management, teamwork and cooperation, problem-solving and decision-making, and situation awareness. An assessor observes the entire team during a procedure and scores individuals in each domain using a 4-point scale ranging from 1 (below standard) to 4 (excellent), with summation of the scores being used to examine the performance of subteams or the team overall (Table 4). A list of behaviours, known as subteam modifiers, rewards positive actions and penalises negative actions, thus influencing scores.

Validity was investigated in the original article by the use of NOTECHS to evaluate surgical teams performing 65 laparoscopic cholecystectomies before (*n* = 26) and after (*n* = 39) teamwork training [16]. Cases were observed by one (*n* = 30), two (*n* = 24), or three (*n* = 11) assessors. Face and content validity could be assumed as the scale had been adapted for surgery in conjunction with expert theatre practitioners. Concurrent validity was established via positive correlation of scores between NOTECHS and the previously validated Safety Attitudes Questionnaire (SAQ). Construct validity was supported by significant improvement of scores after training (*t* = −3.019, *p* = 0.005). OTAS was also used in parallel with NOTECHS to examine convergent validity, which was successfully demonstrated by the excellent agreement between scores (*r* = 0.886, *p* = 0.046).

NOTECHS is a potentially valuable tool due to its detailed analysis of teamwork and subteam modifiers, with recent utilisation to guide the development of a robotic training curriculum [29]. However, aside from the aforementioned study, very little validation evidence of this tool in the surgical setting exists. Further evidence studies from a variety of sources are therefore desired to reinforce validity.

### 4.4. Anaesthetists' Nontechnical Skills (ANTS)

Anaesthetists' Nontechnical Skills (ANTS) is an anaesthetist-specific teamwork assessment tool that was devised from psychological research which identified requisite teamwork skills and structured them into a hierarchal taxonomy based on NOTECHS [30]. Behaviours are examined in 15 elements within four categories: task management, team-working, situation awareness, and decision-making. Exemplar markers are included to guide the assessor in grading the anaesthetist in each element using a 4-point numeric scale ranging from 1 (poor) to 4 (good), with a summary score given for each behavioural category. There is also an option to mark behaviours as “not observed” and each element possesses a comment box for qualitative feedback.

To explore the content validity of the tool, the original designers recruited 50 consultant anaesthetists of varying experience onto a validity study [17]. The practitioners received training in using ANTS and were then asked to rate eight experimental video scenarios with the tool, scoring every element for each scenario. An evaluation questionnaire was also completed. Results showed a high level of content validity as 100% of consultant anaesthetists stated the tool addressed the key behaviours in question, and 84% agreed that no elements appeared to be absent from the tool.

A comprehensive tool encompassing several behavioural aspects, ANTS, has potential value in assessing the NTS of anaesthetists. Analogous to NOTECHS, no other validation evidence exists apart from this study. Construct and criterion validity therefore need to be ascertained before the true value of the assessment can be realised.

### 4.5. Multisource Feedback (MSF)

MSF, or 360° feedback, is a peer assessment tool currently used to review the overall performance of every clinician in the National Health Service (NHS), with results serving as evidence for revalidation. The assessment entails a structured questionnaire designed to relay feedback regarding performance and professional behaviour, which is completed by self-nominated colleagues and patients. Surgical trainees are categorically rated using a 3-point qualitative scale for 16 competencies specified on the form provided by the ISCP, including procedural skills and teamwork.

Face and content validity of MSF in the surgical setting was investigated in a study which recruited 201 surgeons from various specialties who asked 25 consecutive patients to complete the survey, with subsequent analysis of response rates [18]. Face validity was confirmed via endorsement of the included assessment items by the College of Physicians and Surgeons of Alberta, and content validity was established as tool development was based on a list of core nontechnical competencies provided by the surgical committee. The tool has also demonstrated concurrent validity in this field through a study which examined the correlation of scores between MSF and a small-scale combination of an Objective Structural Clinical Examination (OSCE), Direct Observation of Procedural Skills (DOPS), and Internal Medicine In-Training Examination (IM-ITE) [19]. The 209 participants were in their first year of postgraduate residency and a strong positive correlation was observed between the MSF scores and the OSCE + DOPS + IM-ITE scores (*r* = 0.85, *p* < 0.016). However, there is currently no evidence of validity for the ISCP MSF form. There is, however, a lack of evidence supporting construct validity in the surgical setting. Exploration of this area is necessary to provide complete validation, though the MSF tool does appear to be useful due to the variety of feedback sources engaged.

### 4.6. Case-Based Discussion (CbD)

CbD, an adaptation of the valid Chart-Stimulated Recall (CSR) tool [31], is another current instrument which principally evaluates clinical judgement and decision-making. The appraisal involves detailed discussion of a clinical case in the form of a structured interview between the trainee and assessor. Eight domains (including team-working skills) are assessed, with factors such as case complexity accounted for. The trainee is given a 3-point qualitative rating in each domain, a 5-point GSS, and verbal feedback after the discussion.

Surprisingly, very little validation evidence for CbD has been published despite its widespread use in clinical practice, including the ISCP curriculum. Moreover, studies appear to have conflicting conclusions depending on clinical setting. For instance, Foundation Year 1 trainees were found to have increased CbD scores following training progression, demonstrating construct validity [32], whilst CbD assessment of surgical trainees revealed no correlation between scores and training grade, providing evidence against construct validity [33]. This paper also raised concerns about assessor bias, further questioning the validation evidence for this tool.

The value of CbD is questionable due to the insufficient and contradictory validation evidence presented, highlighting the need to conduct further studies and ascertain the validity of this tool in the surgical environment.

### 4.7. Edinburgh Basic Surgical Training Assessment Form (EBSTAF)

The EBSTAF tool was created from a previous list of 70 skills deemed necessary for surgical competence by consultant surgeons using a modified Delphi method [34]. The test encompasses rating of behaviours observed in five domains (communication, knowledge, teamwork, clinical skills, and technical skills) using a 3-point qualitative scale. The assessment forms are completed by various healthcare professionals in multiple departments to provide a comprehensive overview of the trainee's performance.

Validation evidence for EBSTAF is scarce despite having existed for several years. The tool designers presented evidence of construct validity via assessment of 36 surgical trainees using EBSTAF at the beginning and end of the training year [20]. A total of 101 assessments were conducted after a year of training, with results showing a significant improvement in EBSTAF scores across all domains except clinical skills (time 0 to time 1 year median: 81 to 100, *p* = 0.008; 17 to 72, *p* = 0.015; 85 to 100, *p* = 0.018; 82 to 92, *p* = 0.211; 27 to 76, *p* = 0.004). Concurrent validity has also been established in a recent analysis [21].

Although there is great potential in the EBSTAF tool, limited supportive evidence indicates further investigation into the validity of the tool, particularly content validity.

### 4.8. Scrub Practitioners' List of Intraoperative Nontechnical Skills (SPLINTS)

The SPLINTS system is a new teamwork assessment tool for scrub practitioners such as nurses and operating department practitioners [35]. The SPLINTS taxonomy is organised into three skillset categories, each containing three elements against which subjects are marked using a 4-point rating scale. The “situation awareness” category assesses information gathering, information recognition and understanding, and anticipation; “communication and teamwork” contains acting assertively, exchanging information, and coordinating with others; “task management” involves planning and preparation, providing and maintaining standards, and coping with pressure.

As this tool is a recent development, an insufficient amount of time has elapsed to allow for full review of its validity, with only content validity currently established [22]. In the study 34 experienced scrub practitioners completed an evaluation questionnaire following SPLINTS assessment training and practice. Results from the questionnaire showed that 100% of participants agreed that the tool addressed key nontechnical behaviours observed and that no elements listed were unnecessary. 62% and 50% of participants found the tool to be easy to associate observed behaviours with SPLINTS categories and elements, respectively, with 0% and 3% finding it difficult to use the tool. Reliability and internal consistency were also established in this study.

The SPLINTS system shows promise as valuable tool for assessing the teamwork skills of scrub practitioners. However, other forms of validity must be evaluated in order to fully appraise the tool.

### 4.9. Deficiencies in Current Training

As surgery is traditionally viewed as a practical profession, current surgical curricula focus has been towards the development of clinical knowledge and surgical skill, with a distinct absence of formal NTS training [8]. In response to this concern and others regarding current surgical training, an independent inquiry into the Modernising Medical Careers (MMC) training pathway was conducted by Sir John Tooke, who proposed several changes to meet evolving healthcare needs [36]. Recommendations included alteration of the postgraduate training structure from two years of foundation training followed by two years of core specialty training to one year of foundation training followed by three years of core specialty training, with complete abolition of run-through specialist training. The document also comments on the lack of cognitive (NTS) assessment in junior doctor posts.

The final report of another independent review of MMC was authored by Greenaway in 2013 detailing further recommendations to resolve the continual failings of the training pathway, including the necessity to implement NTS training into surgical curricula [37]. Propositions include bringing full GMC registration forward to medical school graduation and the introduction of a formal framework for training teamwork skills outlined in the GMC's* Good Medical Practice* document including communication and leadership training. The former recommendation necessitates that medical students possess some nontechnical competence prior to graduation, indicating the introduction of NTS training into the medical school curriculum. Meier et al. investigated the integration of a simulation-based NTS module adapted from the TeamSTEPPS teamwork training programme into the elective period of the medical school curriculum, with positive results showing a substantial increase in teamwork skills (*p* < 0.001), reinforcing the potential advantage of medical student NTS training [38].

Despite evidence that surgical trainees are safe to operate under direct supervision [39], the increasing patient expectation of surgeons to be technically competent before live operating has instigated significant research into the area of simulation. The benefits of a simulated environment are evident: acquisition of surgical skill without risk of causing harm to the patient, assessment of competence in a controlled reproducible environment, and preparation for crisis scenarios [40]. However, training opportunities with simulation are usually limited and do not form part of the current curriculum. Simulation is an integral part of robotic training curricula, particularly in the field of urology with the modular robotic urology fellowship curriculum devised by the European Robotic Urology Section (ERUS). To date no standardised curriculum exists for such training [41], resulting in varied knowledge and skills between institutions.

There is an increasing awareness of the value of simulation in NTS training and assessment. The feasibility of using a moderate-fidelity simulated operating theatre environment to train surgeons in technical and nontechnical skills simultaneously was explored, with participants confirming the positive immersive experience of realistic simulation environments alongside increased technical skill performance [40]. Similarly, a centralised urological simulation-based program incorporating both technical and nontechnical skills training has been trialled in London, utilising several simulator training materials including laparoscopic and robotic virtual reality simulators and bench top models for technical skills and a high-fidelity simulated operating theatre for NTS [42]. Possessing acceptable face and content validity with a high level of construct validity (*p* < 0.001), realism, acceptability, and feasibility, this dichotomous simulation-based approach may be at the forefront of future surgical training.

### 4.10. Recommendations for Future Training

**Figure 2:**
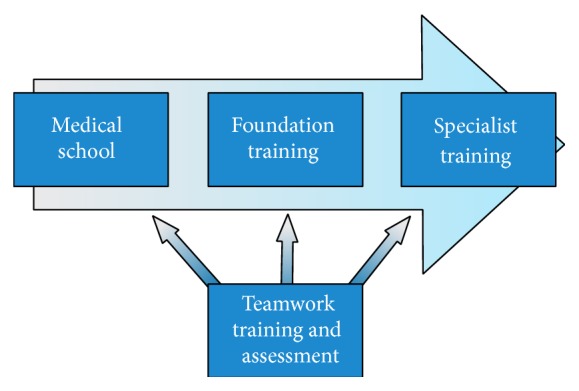
Recommended implementation of teamwork training and assessment.

Based on the current training deficiencies and validation evidence of teamwork assessment tools, the authors suggest the following recommendations to the current UK surgical training curricula:

1. Formal training of the essential NTS that underpin effective teamwork should be assimilated into the medical school curriculum so that graduates possess a basic level of NTS before entering the clinical setting. This training should continue throughout foundation and specialty training (Figure 2).
2. In the current economic climate, high-fidelity simulation should be reserved for senior surgical training as low-fidelity bench models are more cost-effective for training junior trainees [43].
3. Training should also be supplemented with distributed simulation, an inflatable low-fidelity simulator, which may serve as a useful adjunct for junior trainees in encouraging operative confidence between bench model and live operations [44].
4. Concurrent training of technical and nontechnical skills should be a central theme of the surgical curricula.
5. Assessors should receive formal tool training in accordance with national guidelines developed by an expert consensus panel [45].
6. Progression of NTS acquisition should be monitored using the NOTSS teamwork assessment tool.
7. A standardised multistep curriculum should be developed and validated for robotic surgical training with inclusion of NTS assessment.

## 5. Conclusions

Teamwork is a fundamental component of a successful surgical procedure with minimal compromise of patient safety. Current surgical training does not formally encompass development and assessment of the NTS necessary for enhancing team performance. Implementation of NTS training into surgical (or medical school) curricula therefore needs prioritisation to meet the evolving requirements of surgeons and further reduce the occurrence of perioperative adverse events. Training of these skills should ideally be delivered through simulation models to increase skill without risking patient safety, and formal assessment of teamwork must become an integral part of surgical training.

Teamwork assessments are complex tools which focus on evaluating behavioural aspects of each team member. These can be challenging to develop as the tool must possess complex qualities such as the ability to accurately quantify behaviours in a way that is acceptable in practice. A wide range of tools have been discussed and their respective levels of validity established. Currently, NOTSS is the most appropriate sufficiently validated tool to use for teamwork assessment in surgery. Integration of these tools into surgical training is crucial to ensure competent surgeons and safe surgery.

## Figures and Tables

**Table 1:** Teamwork assessment tools and the types of validity established in the surgical environment.

Assessment tool name	Domains assessed	Scoring system	Types of validity established
OTAS	Communication, cooperation, coordination, shared leadership, and team monitoring & situation awareness	7-point Likert scale and generic checklist	Construct [11] and content [12]

NOTSS	Situation awareness, decision-making, communication & teamwork, and leadership	4-point numeric scale	Face [13], content [13, 14], concurrent [15], and construct [13, 14]

NOTECHS	Leadership & management, teamwork & cooperation, problem-solving & decision-making, and situation awareness	4-point numeric scale	Concurrent [16], convergent [16], face [16], content [16], and construct [16]

ANTS	Task management, team-working, situation awareness, and decision-making	5-point numeric scale	Content [17]

MSF	Clinical care, good medical practice, learning & teaching, and teamwork & communication	3-point Likert scale and 3-point GSS	Content [18], face [18], and concurrent [19]

CbD	Medical record keeping, clinical assessment, diagnostic skills, patient management, leadership, clinical judgement, communication & team-working skills, and reflection	3-point Likert scale and 5-point GSS	None^†^

EBSTAF	Communication, knowledge, clinical skills, teamwork, and technical skills	3-point Likert scale	Construct [20] and concurrent [21]

SPLINTS	Communication & teamwork, situation awareness, and task management	4-point Likert scale	Content [22]

OTAS, Observational Teamwork Assessment for Surgery; NOTSS, Nontechnical Skills for Surgeons; NOTECHS, Oxford

Nontechnical Skills; ANTS, Anaesthetists' Nontechnical Skills; MSF, Multisource Feedback; EBSTAF, Edinburgh Basic

Surgical Training Assessment Form; SPLINTS, Scrub Practitioners' List of Nontechnical Skills; GSS, Global Summary Score.

^†^Construct and face validities established in other medical specialties.

**Table 2:** Sources of validation evidence for teamwork assessment tools in surgery.

Study	Assessment tool name	Method of validation	Participants	Types of validity established
Sevdalis et al. [11]	OTAS	Assessment of teamwork during surgical procedures	Surgical trainees, human factors experts, and psychologists	Construct

Hull et al. [12]	OTAS	Content validity metric scoring by expert panel performing surgical procedures	Expert surgeons, nurses, anaesthetists supervisors, anaesthetists, and scrub nurses	Content

Yule et al. [13]	NOTSS	Structured interviews regarding difficult nonroutine cases	Consultant surgeons	Face

Crossley et al. [15]	NOTSS	Assessment of NTS during surgical procedures	Surgical trainees, surgical care practitioners, scrub nurses, and anaesthetists	Concurrent

Beard et al. [14]	NOTSS	Assessment of trainees performing surgical procedures	Surgical trainees, clinical supervisors, anaesthetists, and scrub nurses	Construct and content

Mishra et al. [16]	NOTECHS	Assessment of NTS during laparoscopic cholecystectomies	Surgical trainees, nurses, and anaesthetists	Concurrent, convergent, face, content, and construct

Fletcher et al. [17]	ANTS	Assessment of NTS observed in 8 clinical scenario videos	Consultant anaesthetists	Content

Violato et al. [18]	MSF	Assessment of surgeons using MSF	Surgeons from various specialties and their nominated colleagues	Content and face

Paisley et al. [20]	EBSTAF	Assessment of trainees before and after 1 year of training	Surgical trainees (SHO)	Construct

Mitchell et al. [22]	SPLINTS	Assessment of scrub practitioners in 7 scenarios	Scrub practitioners	Content

OTAS, Observational Teamwork Assessment for Surgery; NOTSS, Nontechnical Skills for Surgeons; NOTECHS, Oxford

Nontechnical Skills; ANTS, Anaesthetists' Nontechnical Skills; MSF, Multisource Feedback; EBSTAF, Edinburgh

Basic Surgical Training Assessment Form; SPLINTS, Scrub Practitioners' List of Intraoperative Nontechnical Skills.

**Table 3:** Nontechnical Skills for Surgeons (NOTSS) scoring form Yule et al.  (2008) [23].

Hospital				
Trainer name				
Date				
Trainee name				
Operation				

Category	Category rating^*∗*^	Element	Element rating^**∗**^	Feedback on performance and debriefing notes

Situation awareness		Gathering information		
		Understanding information		
		Projecting and anticipating future state		

Decision-making		Considering options		
		Selecting and communicating option		
		Implementing and reviewing decisions		

Communication and teamwork		Exchanging information		
		Establishing a shared understanding		
		Coordinating team activities		

Leadership		Setting and maintaining standards		
		Supporting others		
		Coping with pressure		

^*∗*^1 poor; 2 marginal; 3 acceptable; 4 good; NA not applicable.

1 poor: performance endangered or potentially endangered patient safety; serious remediation is required.

2 marginal: performance indicated cause for concern; considerable improvement is needed.

3 acceptable: performance was of a satisfactory standard but could be improved.

4 good: performance was of a consistently high standard, enhancing patient safety; it could be used as a positive example for others.

NA: not applicable.

**(a):** 

Leadership and management	
Leadership	Involves/reflects on suggestions/visible/accessible/inspires/motivates/coaches
Maintenance of standards	Subscribes to standards/monitors compliance to standards/intervenes if deviation occurs/deviates with team approval/demonstrates desire to achieve high standards
Planning and preparation	Team participation in planning/plan shared/understanding confirmed/projects/changes in consultation
Workload management	Distributes tasks/monitors/reviews/tasks prioritised/allots adequate time/responds to stress
Authority and assertiveness	Advocates position/values team input/takes control/persistent/appropriate assertiveness
Teamwork and cooperation	
Team building/maintaining	Relaxed/supportive/open/inclusive/polite/friendly/use of humour/does not compete
Support of others	Helps others/offers assistance/gives feedback
Understanding team needs	Listens to others/recognises ability of team/condition of others considered/gives personal feedback
Conflict solving	Keeps calm in conflicts/suggests conflict solutions/concentrates on what is right
Problem-solving and decision-making	
Definition and diagnosis	Uses all resources/analytical decision-making/reviews factors with team
Option generation	Suggests alternative options/asks for options/reviews outcomes/confirms options
Risk assessment	Estimates risks/considers risk in terms of team capabilities/estimates patient outcome
Outcome review	Reviews outcomes/reviews new options/objective, constructive, and timely reviews/makes time for review/seeks feedback from others/conducts posttreatment review
Situation awareness	
Notice	Considers all team elements/asks for or shares information/aware of available resources/encourages vigilance/checks and reports changes in team/requests reports/updates
Understand	Knows capabilities/cross-checks above/shares mental models/speaks up when unsure/updates other team members/discusses team constraints
Think ahead	Identifies future problems/discusses contingencies/anticipates requirements

Below standard = 1	Behaviour directly compromises patient safety and effective teamwork
Basic standard = 2	Behaviour in other conditions could directly compromise patient safety and effective teamwork
Standard = 3	Behaviour maintains an effective level of patient safety and teamwork
Excellent = 4	Behaviour enhances patient safety and teamwork, a model for all other teams

**Table:** (b) Nontechnical Skills (NOTECHS) subteam modifiers

	Surgical subteam	Anaesthetic subteam	Nursing subteam
Leadership and management			

Positive modifiers	(i) Raises team morale	(i) Takes control when required	(i) Scrub provides clear instructions to circulating nurse(s)
	(ii) Intervenes if deviation occurs	(ii) Demonstrates desire for high standard	(ii) Senior nurse makes sure protocols are followed
	(iii) Prioritises tasks	(iii) Appropriately distributes tasks between rest of team	(iii) Speaks up when unhappy

Negative modifiers	(i) Deflates or fails to motivate team	(i) Does not take control when required	Senior nurse does not support juniors
	(ii) Does not attempt to build cohesion	(ii) Does not set standards	
		(iii) Inappropriate task distribution	

Teamwork and cooperation			

Positive modifiers	(i) Open	(i) Supportive of other subteams	(i) Nurses cooperate and support each other well
	(ii) Appropriate use of abilities within team	(ii) Appreciates functions of other subteams	(ii) Senior nurse covers for junior scrub
	(iii) Supportive of other subteams when necessary		

Negative modifiers	(i) Aggressive in conflicts	(i) Remains idle when problems arise	Poor coordination between equipment needs and those provided
	(ii) Does not appreciate others' abilities	(ii) Functions separately from other subteams	

Problem-solving and decision-making			

Positive modifiers	(i) Demonstrates generation of options	(i) Participates in solving problems	(i) Takes an active part in decision-making
	(ii) Open discussion and agreement over anatomy	(ii) Raises suggestions	(ii) Suggests solutions to problems, for example, alternative equipment
	(iii) Incorporates other subteam issues		

Negative modifiers	(i) Decisions made unsystematically	Does not consider anaesthetic options when faced with problem	Blames the surgeons when faced with problems
	(ii) Does not utilise team where it may benefit		

Situation awareness			

Specific to subteams			

Positive modifiers	Periodically gathers awareness of surroundings	Anticipates surgical and process needs	Anticipates equipment needs

Negative modifiers	Is fixated on operative field	Is not present at important stages of the operation or for long periods of time	Absent at stages when needed to provide service

For all subteams			

Positive modifiers	(i) Patient: has awareness of patient condition/comorbidity		
	(ii) Procedure: appreciates stage of operation		
	(iii) People: who is present in theatre, what skills they have, and what they are doing		
